# Psychometric Properties of the Generalized Anxiety Disorder-7 and Generalized Anxiety Disorder-Mini in United States University Students

**DOI:** 10.3389/fpsyg.2020.550533

**Published:** 2020-09-24

**Authors:** Carol Byrd-Bredbenner, Kaitlyn Eck, Virginia Quick

**Affiliations:** Department of Nutritional Science, Rutgers University, New Brunswick, NJ, United States

**Keywords:** anxiety, psychometrics, generalized anxiety disorder, young adults, instrumental study

## Abstract

**Background:** Generalized anxiety disorder (GAD) is common in young adults, yet few studies have established the psychometric properties of the GAD-7 screener in college students.

**Methods:** A secondary analysis of three studies was conducted to determine GAD-7 factor structure stability, create a GAD-Mini version using standard procedures, and evaluate the psychometric properties, validity, sensitivity, specificity, and predictive values of both versions in young adults.

**Results:** Exploratory and confirmatory principal components analysis indicated the GAD-7 has a single factor structure with strong loadings, reliability, and stability across data collected in three studies. Data from all studies met criteria indicative of good to excellent model fit. Iterative confirmatory principal components analyses revealed the most parsimonious group of items that maintained scale unidimensionality, strong loadings, and high reliability was two items (not able to stop or control worrying and worried too much). Both the GAD-7 and GAD-Mini exhibited good construct and convergent validity. Specificity, sensitivity, and negative predictive value were high, and positive predictive value was moderate to high for the GAD-Mini.

**Conclusions:** The GAD-Mini is a psychometrically sound tool that can serve as a step toward universal screening in clinical practice and contribute to early treatment and improved health outcomes for GAD.

## Introduction

Anxiety is a normal feeling of worry, fear, nervousness, or apprehension that is experienced when facing or anticipating a perceived or real threat (e.g., problem, challenge, and event with an unknown outcome; American Psychiatric Association, 2013). Anxiety disorders, on the other hand, are characterized by persistent, overwhelming worry and fear that interferes with normal functioning (American Psychiatric Association, 2013). The fifth edition of the Diagnostic and Statistical Manual of Mental Health Disorders (DSM-5) includes nine types of anxiety disorders (American Psychiatric Association, 2013). The types are highly comorbid and all are characterized by anxiety, fear, and related behavioral disturbances. The anxiety disorder types differ by the situations, objects, or activities that provoke the anxiety traits.

Generalized anxiety disorder (GAD) is the most common type of anxiety disorder, accounting for nearly 110 million disability days each year in the United States population (Merikangas et al., 2007). GAD is characterized by having excessive worries and fears about non-specific situations, objects, or events on most days for at least 6 months (American Psychiatric Association, 2013). The frequency, intensity, and duration of anxiety and worries are disproportionate to the likely outcome of the actual situation, object, or event. Those with GAD have trouble controlling feelings of worry to the extent that it causes distress or impairment in social, work, and other settings. Symptoms include restlessness or jitteriness, becoming fatigued easily, difficulty concentrating or remembering, irritability, muscle tension, and sleeping problems (Beesdo et al., 2009; American Psychiatric Association, 2013; Wang et al., 2018).

Onset of anxiety disorders is usually in adolescence or early adulthood (de Lijster et al., 2017). The prevalence of anxiety in young adults enrolled in college is higher than the general adult population (LeViness et al., 2017). In fact, results of a 2018 survey of 621 college counseling center directors indicated that anxiety was the main reason students visited campus mental health counseling centers (LeViness et al., 2017). The high prevalence among young adults in college may be a reflection of the transition from adolescence to adulthood characterized by moving into more independent living conditions, increased responsibility for personal decisions, life changes from postsecondary education to university or work life, and having non-family living partners (Arnett, 1997, 2001, 2015).

Anxiety disorders that develop early in life, including young adulthood, are linked with an elevated risk for onset of other mental illness later in life (Shear et al., 2007; Ströhle et al., 2018). Thus, early diagnosis and treatment of anxiety disorders may offer protection against serial comorbidity of other mental illnesses (Ströhle et al., 2018). Brief questionnaires can help healthcare providers rapidly screen patients for a wide array of health conditions, including anxiety disorders. These same questionnaires can serve as self-help tools that can be delivered to consumers conveniently, economically, and privately by the Internet (Donker et al., 2011; Lee and Kim, 2019). Access to health screening tools can provide individualized feedback that may encourage individuals with GAD symptoms to seek treatment from a professional.

Ideally, health screeners are brief, easy to administer and score, and address key diagnostic criteria. The Generalized Anxiety Disorder scale (GAD-7) is a widely used 7-item, 4-point Likert scale designed to screen for GAD according to DSM criteria (Spitzer et al., 2006). The time constraints of doctor office visits along with the desire for rapid completion and feedback to consumers contributed to the development of the 2-item GAD-2. The psychometric properties of the GAD-7 and GAD-2 are reported to be good to excellent in an array of studies examining these questionnaires (Plummer et al., 2016). For example, psychometric studies have included the general population in The Netherlands (Donker et al., 2011), Brazil (Silva et al., 2018), and Germany (Löwe et al., 2010; Wild et al., 2014; Hinz et al., 2017); teens in Finland (Tiirikainen et al., 2019); a rural population in China (Luo et al., 2019); patients receiving addictions treatment in the United Kingdom (Delgadillo et al., 2012); primary care patients in Spain (García-Campayo et al., 2012), United States (Kroenke et al., 2007), Finland (Kujanpaa et al., 2014), and Malaysia (Sidik et al., 2012); psychiatric patients in Australia (Staples et al., 2019), United States (Kertz et al., 2013), Spain (Cano-Vindel et al., 2018), and Turkey (Konkan et al., 2013); patients receiving epilepsy care in France (Micoulaud-Franchi et al., 2017); patients with migraine in Korea (Seo and Park, 2015); patients with cancer in Germany (Esser et al., 2018); patients with multiple sclerosis in the United States (Hughes et al., 2018); pregnant women in the United States (van Heyningen et al., 2018), United Kingdom (Nath et al., 2018), and Ghana and Cote d’Ivoire (Barthel et al., 2014); individuals receiving care in a traditional medicine department in China (Zeng et al., 2013); and those speaking an array of languages (Donker et al., 2011; García-Campayo et al., 2012; Sidik et al., 2012; Sousa et al., 2015; Ahmad et al., 2017; Ahn et al., 2019).

Despite the prevalence of anxiety in young adults and the critical importance of understanding how this measure performs in this population, the only psychometric studies of the GAD questionnaires that could be located for this age group were for university students in Korea (Lee and Kim, 2019) and Portugal (Bartolo et al., 2017). Both of these studies found a single factor model of the GAD-7 to be appropriate for use in college student samples. Although the GAD-2 has been used in several studies, no published data could be located using the standard procedures for shortening instruments; rather, two items were identified as “core” items and used to truncate the GAD-7 questionnaire to two items (Kroenke et al., 2007; Skapinakis, 2007). Further, no study could be located that examined the stability of the psychometric properties of the GAD-7 and shortened versions of this questionnaire at different points in time with a defined audience (i.e., individuals in the life stage of young adulthood who are enrolled in college). Hence, the goal of this study was to conduct a secondary analysis of three large cross-sectional studies conducted at time points ranging from 2009–2010 to 2018–2019 to determine the stability of the factor structure of the GAD-7 in the target audience over time, create a GAD-Mini version of this questionnaire using standard procedures, evaluate the psychometric properties of both versions, assess construct and convergent validity, and determine sensitivity, specificity, and predictive values for identifying GAD in free-living (non-institutionalized) young adults enrolled in a university in the United States.

## Materials and Methods

These studies were approved by the Institutional Review Board at the authors’ university. Participants in all studies gave informed consent.

### Sample

Individuals were recruited *via* verbal and electronic announcements to participate in an online survey of health practices of young adults. Data for this secondary analysis were collected in three separate, unrelated cross-sectional studies conducted in 2009–2010, 2015, and 2018–2019 (Quick, 2011; Byrd-Bredbenner et al., 2016; Eck, 2020, unpublished), labeled as Data Set A, B, and C, respectively. To be eligible, participants had to be enrolled at Rutgers, The State University of New Jersey, United States and be young adults (i.e., 18–26 years of age).

### Study Measures

Participants in all three studies completed an online survey that gathered demographic data (e.g., age, gender, and race/ethnicity) and included the 2-item Patient Health Questionnaire (PHQ-2) and GAD-7 questionnaire. The PHQ-2 is a commonly used reliable, valid questionnaire that screens for Major Depressive Disorder (MDD) according to the criteria in the DSM-5 (Kroenke et al., 2003; Spitzer et al., 2006). The PHQ-2 assesses the frequency of depressive symptoms over the past 2 weeks. PHQ-2 items are scored on a 4-point Likert scale (0 = not at all to 3 = nearly every day); summed item scores create the total scale score with a possible score range of 0–6 with higher scores indicating greater depression severity. The 7-item GAD-7 questionnaire examines frequency of anxiety symptoms, such as worrying and feeling nervous and has the same answer choices as the PHQ-2 (Spitzer et al., 2006). The GAD-7 scale score is determined by summing individual item scores. Total scores can range from 0 to 21, with a score of ≥10 indicating the threshold for GAD per DSM-V criteria (Spitzer et al., 2006).

### Data Analysis

Data were analyzed using SPSS and AMOS (a SPSS module) version 26.0 (SPSS Inc., Chicago, IL, United States). Kaiser-Meyer-Olkin (KMO) testing was conducted separately for each of the data sets to assess sampling adequacy, with values closer to 1 indicating greater adequacy of sampling (Glen, 2020). Each data set was independently subjected to Bartlett’s test of sphericity to confirm the GAD items were related and suitable for factor analysis; significance for this test was set at *p* < 0.01 (Glen, 2020; IBM Knowledge Center, 2020).

To identify the factor structure of the GAD items, exploratory principle components analysis with orthogonal (varimax) and oblique (oblimin) rotations were conducted separately with all three Data Sets (Harlow, 2014). All items were allowed to freely load to determine the number of factors present. Subsequently, all Data Sets were independently subjected to confirmatory principal component analysis to verify the factor structure (i.e., by specifying number of factors) identified from the exploratory principal components analysis (Osborne, 2014; Flora and Flake, 2017). The confirmatory principal components analysis results for all Data Sets were compared to determine whether the items loaded on the same factors in all three Data Sets. Then, the magnitude of difference in each item’s factor loading from the confirmatory principal components analysis was compared across Data Sets. Magnitude of difference was calculated by squaring the difference between the factor loading for corresponding items in Data Set A and Data Set B, Data Set A and Data Set C, and Data Set B and Data Set C. Magnitude differences of ≤0.05 were considered to be small and indicative of a stable factor structure (Osborne, 2014).

Using SPSS AMOS, goodness-of-fit indicators examined comparative fit index (CFI), goodness-of-fit index (GFI), Tucker-Lewis Index (TLI), root mean square error of approximation (RMSEA) and related 90% confidence interval (CI), and standardized root mean-square residual (SRMR; Hu and Bentler, 1999; Schermelleh-Engel et al., 2003; Harlow, 2014; Parry, Undated). The values for all of these indicators ranged from 0 to 1, with higher values indicating better fit for CFI, GFI, TFI, and RMSEA and values near 0 indicating better fit for SRMR (Schermelleh-Engel et al., 2003). Chi-square (*χ*^2^) and degrees of freedom (*df*) also were calculated but due to the large sample sizes could not be considered as absolute fit indicators (Schermelleh-Engel et al., 2003; Boateng et al., 2018).

After the factor structure was determined and verified, the next step was to reduce the length of the GAD-7 to items to create a shortened version of the questionnaire, the “GAD-Mini.” The item elimination strategy considered factor loadings that were cross (>0.40) and/or weak (<0.60; Harlow, 2014). In cases where the lowest loading item exceeded these *a priori* cross and/or weak loading thresholds, the lowest loading item was eliminated. Internal consistency (Cronbach alpha) was computed after each item elimination to ensure acceptable (>0.70) but not excessive (>0.85) reliability, an indicator of item redundancy (Streiner, 2003; Tavakol and Dennick, 2011). After each item elimination, the factors also were reviewed for logical sense to ensure content validity. The goal was to iteratively continue eliminating the lowest loading item, one at a time, until all remaining items retained strong loadings and yielded an acceptable Cronbach alpha coefficient.

Research indicates that anxiety is more common in women (Scheibe and Albus, 1992; Yonkers et al., 2003; Vesga-Lopez et al., 2008). Thus, construct validity was determined by comparing total scores on the GAD-7 and the GAD-Mini by sex using independent, 2-tailed *t*-tests (Löwe et al., 2010). Convergent validity was assessed by comparing a measure known to be related to anxiety using Spearman rank-order correlations. The related measure was depression as measured by the PHQ-2 (Kroenke et al., 2003).

Spearman rank-order correlations were used to determine agreement between the GAD-7 and GAD-Mini scores in the assessment of sensitivity (to accurately identify anxiety), specificity (to accurately identify an individual as not having anxiety symptoms), positive predictive value (PPV; the probability of a positive diagnosis after a positive screening), and negative predictive value (NPV; the probability of a negative diagnosis after a negative screening). To calculate sensitivity, specificity, positive PPV, and NPV, the total GAD-7 score and total GAD-Mini score were dichotomously coded to indicate whether the participant was above or below the previously established threshold with greatest sensitivity and specificity indicative of anxiety [i.e., ≥10 of the possible 21 points on the GAD-7 (Spitzer et al., 2006), which is an equivalent of 48% of the total possible points]. The threshold for GAD-Mini was set at 48% of the total points possible, e.g., a 2-item GAD-Mini would have a possible score range of 0–6, thus the threshold equaled (≥6 × 0.48). Sensitivity, specificity PPV, and NPV of the GAD-Mini were all calculated using the GAD-7 as the “gold” standard (Parikh et al., 2008; Plummer et al., 2016; Hinz et al., 2017; Silva et al., 2018; Luo et al., 2019).

## Results

Table 1 summarizes the demographic characteristics of the participants in the three studies. Participants’ average age ranged from 19.64 to 20.45 years. Most were women and the proportion who were white declined over time from 58% in Data Set A to 41% in Data Set C.

**Table 1:** Demographic characteristics of participants in all studies.

Characteristic	Data Set A Study 2009–2010	Data Set B Study 2015	Data Set C Study 2018–2019
*N*	1,897	426	1,805
Age (Mean + SD)	19.60 ± 1.62	20.19 ± 1.82	20.44 ± 1.47
*N* (%) White	1,065 (56)	204 (48)	748 (41)
*N* (%) Women	1,081 (57)	274 (64)	1,172 (65)

KMO calculations revealed that all three data sets met the criteria for sampling adequacy (Glen, 2020). The KMO values were 0.92, 0.91, and 0.93 for Data Sets A, B, and C, respectively. Bartlett’s test of sphericity values were significant (*p* < 0.0001) for Data Sets A, B, and C (i.e., *χ*^2^ = 7,622.071, 1,983.27, and 9,598.23, respectively); therefore, the GAD-7 items were related and suitable for factor analysis (Glen, 2020; IBM Knowledge Center, 2020).

For Data Set A, the exploratory principal components analysis for all seven items yielded one eigenvalue >1, thus indicating a single factor solution (Harlow, 2014). Additionally, rotations were not possible because only one factor was extracted, indicating a unidimensional scale. Principal components analysis procedures indicated factor structure replicability in that both Data Sets B and C also yielded a single factor solution. Similarly, confirmatory principal components analysis revealed the same single factor structure and similar loadings for all Data Sets (Table 2). As shown in Table 2, the magnitude of difference for each item’s factor loading between Data Sets A and B ranged from 0.000 to 0.007, 0.000 to 0.003 for Data Sets A and C, and 0.000 to 0.003 for Data Sets B and C. The magnitude of differences for all factor loading comparisons were well below the threshold of ≤0.05 and were indicative of a stable factor structure (Osborne, 2014).

**Table 2:** Principal components analysis factor loadings and magnitude of differences^a^ items for the Generalized Anxiety Disorder-7 (GAD-7) Questionnaire for Data Sets A, B, and C.

Data Set	Factor loadings						
	GAD #1^b^	GAD #2	GAD #3	GAD #4	GAD #5	GAD #6	GAD #7
	How often have you felt nervous, anxious, or on edge?	How often have you not been able to stop worrying or control worrying?	How often have you worried too much about different things?	How often have you had trouble relaxing?	How often have you been so restless that it is hard to sit still?	How often have you become easily annoyed or irritable?	How often have you felt afraid as if something awful might happen?
A (*n* = 1,897)	0.820	0.840	0.849	0.854	0.739	0.733	0.780
B (*n* = 426)	0.813	0.874	0.872	0.858	0.735	0.814	0.765
C (*n* = 1,805)	0.863	0.896	0.887	0.871	0.792	0.758	0.806
**Magnitude of Differences**							
A vs. B	0.000	0.001	0.001	0.000	0.000	0.007	0.000
A vs. C	0.002	0.003	0.001	0.000	0.003	0.001	0.001
B vs. C	0.003	0.001	0.000	0.000	0.003	0.003	0.002

a Magnitude of differences is the squared difference in factor loading coefficient between two Data Sets.

b Generalized Anxiety Disorder (GAD) item #.

Goodness-of-fit findings are shown in Table 3. CFI, GFI, TLI, and SRMR values indicate good to excellent model fit for all Data Sets (Schermelleh-Engel et al., 2003). RMSEA for Data Set A was acceptable and was somewhat exceeded the generally recommended acceptable fit cutoff (≤0.10) in Data Sets B and C (Hu and Bentler, 1999).

**Table 3:** Goodness-of-fit indices for GAD-7 Questionnaire for Data Sets A, B, and C.

Statistic	Data Set A (*n* = 1,897)	Data Set B (*n* = 426)	Data Set C (*n* = 1,805)
*χ*^2^ (*df*)^a^	181.20 (14)^*^	107.80 (14)^*^	344.56 (14)^*^
CFI	0.978	0.953	0.966
GFI	0.972	0.924	0.940
TLI	0.967	0.929	0.948
RMSEA (90% CI)	0.079 (0.069 to 0.090)	0.126 (0.104 to 0.148)	0.114 (0.104 to 0.125)
SRMR	0.026	0.038	0.035

* *p* < 0.0001.

a Df, degrees of freedom; CFI, comparative fit index; GFI, goodness-of-fit index; TLI, Tucker-Lewis Index; RMSEA, root mean square error of approximation; CI, confidence interval; and SRMR, standardized root mean-square residual.

Table 4 reports the iterative confirmatory principal components analyses conducted to reduce the number of items in the GAD-7 to the most parsimonious set of items. Each iteration of the confirmatory principal components analysis generated virtually identical findings to the first iteration; that is, there was one eigenvalue >1, no items had cross loadings or weak loadings, the scale was unidimensional, and the Cronbach alpha coefficients were high. Per the data analysis plan, the lowest loading item in Data Set A was eliminated and confirmatory principal components analysis was repeated. For Data Set A, iterations could continue per the analysis plan until only two GAD items remained. Repeating these procedures with Data Sets B and C yielded similar findings, resulting in only two GAD items remaining. Thus, the GAD-Mini was comprised of item 2 (How often have you not been able to stop worrying or control worrying?) and item 3 (How often have you worried too much about different things?) from the GAD-7. Cronbach alpha coefficients remained strong after every iteration.

**Table 4:** Iterative confirmatory principal components analysis factor loadings for the GAD Questionnaire items for Data Sets A, B, and C.

Data Set	PCA iteration	Factor loadings							Cronbach alpha
		GAD #1^a^	GAD #2	GAD #3	GAD #4	GAD #5	GAD #6	GAD #7	
A (*n* = 1,897)	1	0.820	0.840	0.849	0.854	0.739	0.733	0.780	0.91
	2	0.829	0.851	0.857	0.855	0.745		0.784	0.90
	3	0.837	0.865	0.877	0.847			0.791	0.90
	4	0.845	0.878	0.889	0.860				0.89
	5		0.895	0.907	0.875				0.87
	6		0.931	0.931					0.85
B (*n* = 426)	1	0.813	0.874	0.872	0.858	0.735	0.814	0.765	0.92
	2	0.828	0.889	0.888	0.846		0.810	0.770	0.92
	3	0.831	0.899	0.901	0.855		0.813		0.91
	4	0.841	0.914	0.918	0.854				0.90
	5	0.926	0.929	0.879					0.90
	6		0.955	0.955					0.90
C (*n* = 1,805)	1	0.863	0.896	0.887	0.871	0.792	0.758	0.806	0.93
	2	0.877	0.908	0.899	0.874	0.788		0.801	0.93
	3	0.889	0.919	0.913	0.869			0.803	0.93
	4	0.902	0.926	0.924	0.876				0.93
	5	0.921	0.938	0.932					0.92
	6		0.955	0.955					0.90

a Generalized Anxiety Disorder (GAD) item #.

Construct validity was confirmed with independent two-tailed *t*-tests showing that anxiety scores were significantly higher in women than men on both the GAD-7 and GAD-Mini for all three Data Sets (Table 5). Similarly, convergent validity was confirmed with Spearman rank-order correlations showing that PHQ-2 and GAD-7 were moderately correlated as were PHQ-2 and the GAD-Mini (Table 6).

**Table 5:** Examining gender differences of GAD-7 and GAD-Mini to establish construct validity.

Data Set	GAD-7			GAD-Mini		
	MenN Mean ± SD	WomenN Mean ± SD	p^*^	MenN Mean ± SD	WomenN Mean ± SD	p^*^
A	*N* = 816 4.84 ± 4.64	*N* = 1,081 6.57 ± 5.21	<0.0001	*N* = 816 1.50 ± 1.60	*N* = 1,081 2.17 ± 1.80	<0.0001
B	*N* = 152 5.99 ± 4.86	*N* = 274 8.34 ± 5.94	<0.0001	*N* = 152 1.88 ± 1.69	*N* = 274 2.73 ± 2.01	<0.0001
C	*N* = 633 6.09 ± 5.42	*N* = 1,172 8.81 ± 6.08	<0.0001	*N* = 633 1.86 ± 1.83	*N* = 1,172 2.78 ± 1.97	<0.0001

* Two-tailed independent *t*-tests comparing gender differences of GAD-7 and GAD-Mini.

**Table 6:** Spearman rank-order correlations to establish convergent validity of (GAD-7) and GAD-Mini with Patient Health Questionnaire (PHQ-2) depression severity scores.

Measure	Spearman rank-order correlations		
	Data Set A (*n* = 1,897) PHQ-2	Data Set B (*n* = 426) PHQ-2	Data Set C (*n* = 1,805) PHQ-2
GAD-7	0.65	0.45	0.64
GAD-Mini	0.58	0.39	0.57

Table 7 indicates that the GAD-7 and GAD-Mini scores were highly correlated (*R* ≥ 0.90). At the threshold of ≥3, sensitivity of the GAD-Mini to accurately identify anxiety and specificity of the GAD-Mini to accurately identify an individual as not having anxiety symptoms was high across Data Sets. Additionally, nearly all young adults who were identified by the GAD-Mini as non-cases of GAD (NPV) were also classified as such by the GAD-7; however, only 59–75% of those having GAD identified by the GAD-Mini (PPV) were similarly classified by the GAD-7.

**Table 7:** Correlation, specificity, sensitivity, and predictive value of GAD scores.

Data Set	Spearman rank-order correlation of total GAD-7 and total GAD-Mini scores	Specificity of GAD-Mini^*^	Sensitivity of GAD-Mini^*^	Positive predictive value	Negative predictive value
A (*n* = 1,897)	0.90	0.86	0.94	0.59	0.99
B (*n* = 426)	0.92	0.82	0.98	0.64	0.99
C (*n* = 1,805)	0.92	0.86	0.94	0.75	0.97

* Calculated with GAD-7 as the “gold” standard; threshold indicating GAD was ≥10 for GAD-7 and ≥3 for GAD-Mini.

## Discussion

Exploratory and confirmatory principal components analysis results of this study indicate that the GAD-7 has a single factor structure with strong loadings, high reliability coefficient, and stability in young adults enrolled in college over time. All Data Sets met *a priori* criteria indicative of good to excellent model fit except the RMSEA criterion in Data Sets B and C. Iterative confirmatory principal components analyses revealed the most parsimonious set of items that maintained scale unidimensionality, strong loadings, and high reliability was two items (not able to stop worrying or control worrying and worried too much about different things); this finding was stable across all three sets of data. Both the GAD-7 and GAD-Mini exhibited good construct and convergent validity in all three data sets. In every data set, the specificity, sensitivity, and NPV was high and PPV was moderate to high for the GAD-Mini.

The unidimensionality and Cronbach alpha coefficients of the GAD-7 found in this study are congruent with studies conducted in other populations. For example, this study had strikingly analogous findings to those reported for Korean and Portuguese university students (Bartolo et al., 2017; Lee and Kim, 2019). Similar findings also were reported for primary care patients (Jordan et al., 2017; Johnson et al., 2019), psychiatric patients (Dear et al., 2011; Kertz et al., 2013; Konkan et al., 2013; Rutter and Brown, 2017; Johnson et al., 2019), infertility patients (Omani-Samani et al., 2018), the general population in several countries (Konkan et al., 2013; Sousa et al., 2015; Hinz et al., 2017), adolescents (Tiirikainen et al., 2019), individuals receiving care in a traditional medicine department in China (Zeng et al., 2013), and in non-English speakers (Mills et al., 2014; Sousa et al., 2015; Ahmad et al., 2017). Further, the goodness-of-fit findings parallel those reported for populations with varying characteristics (García-Campayo et al., 2012; Mills et al., 2014; Bartolo et al., 2017; Hinz et al., 2017; Rutter and Brown, 2017; Doi et al., 2018; Lee and Kim, 2019).

To the best of the authors’ knowledge, this is the first study to conduct iterative factor analysis to shorten the GAD-7 to the most parsimonious set of items yielding strong psychometric properties. Although the GAD-2 was proposed in 2007 (Kroenke et al., 2007; Skapinakis, 2007), the two items comprising this measure were chosen because they were considered the “core” items (i.e., items 1 and 2 on the GAD-7: How often have you felt nervous, anxious, or on edge? and How often have you not been able to stop worrying or control worrying?). All three data sets in this study yielded two items from the GAD-7, with one item being the same as the GAD-2 (i.e., item 2: How often have you not been able to stop worrying or control worrying?) and the other differing (i.e., item 3: How often have you worried too much about different things?). The key difference seems to be that GAD-Mini focuses on excessive, difficult to control worry – the primary feature of GAD, whereas the GAD-2 combines worry with one of the six symptoms associated with anxiety and worry (i.e., feeling on edge; American Psychiatric Association, 2013). Given that there are five other symptoms (i.e., fatigue, difficulty concentrating, irritability, muscle tension, and sleep disturbances) it may be that the single symptom targeted in item 1 of the GAD-7, insufficiently represented the possible array of GAD symptoms. Interestingly, Donker et al. (2011) observed in a Dutch general population that it was not the GAD-7 “core” items 1 and 2 that explained the most variance in the GAD-7 scale – it was item 4 of the GAD-7 (i.e., How often have you had trouble relaxing?). This study along with that of Donker et al. (2011) suggest that future research directions should include the re-analysis of existing GAD-7 data sets using iterative confirmatory principal components analysis to confirm the psychometric properties of the GAD-Mini in other population groups and to determine its utility in relation to the GAD-2.

The GAD-7 and GAD-Mini both exhibited good construct and convergent validity, thereby supporting an array of previous studies. For example, congruent with other research (Ahmad et al., 2017; Hinz et al., 2017), women scored significantly higher on the full length and shortened measures than men. In addition, the moderate correlation between both measures and PHQ-2 was also demonstrated in other studies (Löwe et al., 2010; García-Campayo et al., 2012; Wild et al., 2014; Bartolo et al., 2017; Hinz et al., 2017; Omani-Samani et al., 2018; Ahn et al., 2019; Lee and Kim, 2019).

Like reported GAD-7 and GAD-2 comparisons (Kroenke et al., 2007; Donker et al., 2011; Delgadillo et al., 2012; Hinz et al., 2017; Hughes et al., 2018), the GAD-Mini was highly correlated with the GAD-7 suggesting that these measures may be equally effective in screening for GADs. In addition, the threshold score of ≥10 for the GAD-7 that was identified in previous research (Spitzer et al., 2006) and ≥3 score for the GAD-Mini used previously for the GAD-2 (Kroenke et al., 2007, 2010), resulted in near perfect agreement between the GAD-Mini and GAD-7 in identifying those above and below the threshold indicative of GAD. At the threshold of ≥3, the GAD-Mini correctly identified nearly all GAD cases (sensitivity) and more than eight in 10 of the non-cases of GAD (specificity). Although the GAD-Mini performs similarly to the GAD-7 in identifying those without GAD (NPV), the rate at which it classified those with GAD is higher than the GAD-7 (PPV). This finding suggests that when the GAD-Mini indicates GAD, it would be wise to confirm this with the longer GAD-7 to more clearly pinpoint symptoms of anxiety and, given its wider range of possible scores compared to the GAD-Mini (0–21 vs. 0–6), for tracking changes over time in clinical practice as treatment progresses (Kroenke et al., 2007; Hinz et al., 2017).

This study’s findings are limited to young adults enrolled at a large United States university, however the sample in all data sets was large and students were racially diverse. Additionally, study findings parallel those of university students in Portugal (Bartolo et al., 2017) and Korea (Lee and Kim, 2019) suggesting generalizability across this population. A further limitation is that this study relied on the GAD-7 to serve as the gold standard. Interviews by a psychologist or psychiatrist would have been a true gold standard (Baker et al., 2018), however this was not feasible given the online data collection method and size of the sample recruited. Moreover, the GAD-7 repeatedly has been found to have good to excellent psychometric properties for identifying GAD and has adequately served as the gold standard in similar studies (Löwe et al., 2010; Plummer et al., 2016; Hinz et al., 2017). Like all secondary data analyses, this study is limited to existing data, however the original data were carefully collected, cleaned, and stored and contained complete data for all participants. Importantly, possible effects caused by size, location, and/or mission of the higher education institution were controlled for by including only data collected from students at one university. An important strength of this study is that, to the authors’ knowledge, it is the first study to establish the psychometric properties of the GAD-7 and GAD-Mini in United States university students and demonstrate its stability in diverse young adults enrolled in college over a time period spanning a decade.

The GAD-Mini addresses the primary feature of GAD, namely worry. Its excellent psychometric qualities, along with the speed with which the GAD-Mini can be completed and scored, make it a valuable screener for practitioners and as a self-help tool for young adults. The ease with which it can be used has the potential for facilitating early diagnosis of a condition prevalent among young adults enrolled in college (LeViness et al., 2017). Left undiagnosed and untreated, GAD can become chronic, resistant to treatment, and increases the risk for comorbidity with other mental illness, such as depression (Shear et al., 2007; Maron and Nutt, 2017; Ströhle et al., 2018). Despite the high rates of GAD in older teens and young adults, the United States Preventive Services Task Force and American Academy of Pediatrics have not recommended screening for anxiety. The GAD-Mini is a psychometrically sound screening tool that can serve as a step toward universal screening in even the busiest clinical practice and contribute to early treatment and improved health outcomes in those with GAD.

## Data Availability Statement

The raw data supporting the conclusions of this article will be made available by the authors, without undue reservation.

## Ethics Statement

The studies involving human participants were reviewed and approved by Rutgers University Institutional Review Board. The patients/participants provided their written informed consent to participate in this study.

## Author Contributions

All authors were responsible for study design, statistical analysis, and manuscript preparation. CB-B and VQ were responsible for recruiting the participants for Data Set A. CB-B and KE were responsible for recruiting the participants for Data Sets B and C. All authors have contributed to and have approved the final manuscript.

### Conflict of Interest

The authors declare that the research was conducted in the absence of any commercial or financial relationships that could be construed as a potential conflict of interest.
